# Datasets on factors influencing trading on pedestrian bridges along Ikorodu road, Lagos, Nigeria

**DOI:** 10.1016/j.dib.2018.06.055

**Published:** 2018-06-22

**Authors:** Olabisi O. Ajakaiye, Hammed A. Afolabi, Adedotun O. Akinola, Hilary I. Okagbue, Omoniyi O. Olagunju, Olufumilayo O. Adetoro

**Affiliations:** a Department of Urban and Regional Planning, Yaba College of Technology, Lagos, Nigeria; b Department of Architecture, Covenant University, Canaanland, Ota, Nigeria; c Department of Mathematics, Covenant University, Canaanland, Ota, Nigeria

**Keywords:** Likert scale, Survey analytics, Pedestrian bridges, Lagos, Transportation, Environment

## Abstract

The survey data was obtained from a study that investigated factors responsible for the patronage of the traders on the pedestrian bridges along Ikorodu road, Lagos state, Nigeria. Survey research was adopted for this investigation while data were primarily sourced. The sample frame adopted for this study was the average total number of people using the pedestrian bridges per day along Ikorodu road was estimated as 240,380, while the sample size was 384, based on Cochran׳s sample size formula. The convenience, non-probability sampling technique was used for the survey. Data were analyzed using descriptive statistics (frequency tables) and inferential statistics techniques (factor analysis for data reduction and categorization, communalities of variables and KMO) while Likert scale was used as a means of measurement. The datasets can be considered in the commerce and environmental policies of Lagos State and Nigeria with a view to recommending policies that will encourage easy movement of people and the effective uses of the transport facilities.

**Specifications Table**

**Table:** 

Subject area	Environmental Science
More specific subject area	Transportation Management
Type of data	Tables and Figures
How data was acquired	Field Survey in some selected pedestrian bridges along Ikorodu road, Lagos, Nigeria
Data format	Raw and analyzed
Experimental factors	Simple percentages and level of agreed index (LAI) were used as analytical tool of the generated data. Factor analysis was used in determining the factors influencing the patronage of the traders on pedestrian bridges. Likert scale also ranked factors using the Sum of weighted values (SWV).
Experimental features	The key method used in data collection - structured questionnaire designed in Likert scale, the questionnaire was designed in such a way that it helped to collate basic information from the respondents. A population size of two hundred and forty thousand three hundred and eighty (240,380) was selected, and a total sample size of 384 respondents was used in data generation, with questionnaire distributed to pedestrian bridge users. Variables pertaining to the above listed targets. 14 samples were excluded because of non-response.
Data source location	Ikorodu road, Lagos, Nigeria
Data accessibility	All the data are in this data article

**Value of the data**

• The data can be used to review of Lagos State transportation, commerce,environmental policies.
• The dataset can also be for safety and precautionary measures on pedestrian bridges in Lagos and other major cities across Nigeria.
• The data can be used for educational and research purposes.
• The questionnaire for this survey can be adopted and modified to include subjects not included in this article.

## 1 Data

The data in this article was obtain from a field survey aimed at the determination of perceived factors that influences pedestrian in patronizing traders on pedestrian bridges along Ikorodu road in Lagos, Nigeria. Trading on the pedestrian bridges is a subset of the phenomenon known as “street trading” or street hawking”. The pedestrian bridges are constructed on major expressways to ease transportation. Over the years, the pedestrian bridges have become a place where business transactions are conducted between traders and pedestrian, even though that street trading is outlawed in the Lagos metropolis. The data collected on the factors that encourage such business transactions are presented in this article. The socio-demographics of the respondents are presented in Table 1, Table 2, Table 3, Table 4, Table 5, Table 6.

**Table 1:** Sex of the respondents.

Sex	Frequency	Percentage
Male	170	45.9
Female	200	54.1
Total	370	100

**Table 2:** Age of the respondents.

Ages (yrs)	Frequency	Percentage
10–20	44	11.9
21–40	264	72.8
41–60	62	15.3
61and above	0	0
Total	370	100

**Table 3:** Marital status of the respondents.

Marital status	Frequency	Percentage
Single	148	40.0
Married	214	57.8
Divorced	8	2.2
Widow/widower	0	0
Total	370	100

**Table 4:** Religion of the respondents.

Religion	Frequency	Percentage
Christianity	208	56.2
Islam	162	43.8
African tradition	0	0
Total	370	100

**Table 5:** Level of education attained by the respondents.

Educational background	Frequency	Percentage
Primary	95	25.7
Secondary	150	40.5
BSc/ HND	113	30.6
Informal training	12	3.2
Total	370	100

**Table 6:** Level of monthly Income (Nigerian Naira) of the respondents.

Monthly income	Frequency	Percentage
Below #10,000	84	22.7
#11,000 - #20,000	88	23.8
#21,000 - #30,000	92	24.9
#31,000 and above	106	28.6
Total	370	100

Subsequently, several aspects of trading on pedestrian bridges or similar phenomena can be explored. Some of them are outlined: child trading on pedestrian bridges, incidence of robbery on pedestrian bridges, epidemiology of injuries that occurred on pedestrian bridges, the menace of alms begging on pedestrian bridges, prostitution on pedestrian bridges, the economic benefits of trading on pedestrian bridges, poverty, unemployment and illiteracy as predictors of trading on pedestrian bridges and others. Some of these have been researched as street trading or street hawking [1], [2], [3], [4], [5], [6], [7], [8], [9], [10]. Trading on the pedestrian bridges and street trading in general are part of social problems facing the Lagos metropolis. Others are transportation using bus rapid transit [11], crime [12]**,** gambling [13], housing, construction and estate management [14], [15], [16], [17], [18], power outages [19], [20], water, sanitation, waste management and hygienic issues [21], [22], [23], [24], prostitution, sexual violence, HIV incidence and drug abuse [25], [26], [27], [28] and unemployment [29]. In addition, other statistical analysis can be applied such as in [30], [31], [32], [33], [34], [35], [36], [37], [38], [39], [40].

In summary, data revealed that young adults (21–40 years), female and married persons were the people mostly patronizing the traders on the surveyed bridges.

## 2 Experimental design, materials and methods

The study area (pedestrian bridges along Ikorodu road, Lagos, Nigeria) was chosen because the road linked to several cities in the metropolis and pedestrian bridges located there often experience heavy pedestrian movement. Also the bridges are the only means of crossing from one part of the expressway to another since pedestrian crossing on the expressway is outlawed. The traders often use the avenue of heavy movement of people on the pedestrian bridges to display their wares and solicit sales from the people. On the other hand, disable people are often seen on the bridges begging for alms.

The sample frame adopted for this study was the average total number of people using the pedestrian bridges per day along Ikorodu road was estimated as 240,380, while the sample size was 384, based on Cochran׳s sample size formula.

The convenience sampling which is a non-probability sampling technique was adopted for the survey because most of the respondents were interviewed by circumstantial-convenience. This sampling technique was very beneficial because the survey was done in the evening when people are returning from work, schools, markets, offices or shops. The morning was not used because the pedestrian are rushing to work and may not have time to complete the questionnaires.

Factor analysis was used to analyze the data. Results of factor analysis for pedestrians’ perceived factors of pedestrian bridge trading patronage revealed a K.M.O. value of 0.618 with Bartlett׳s test significance level of 0.000 presented in Table 7. The result of tests implies that the data is suitable for factor analysis.

**Table 7:** KMO and Bartlett׳s Test.

Kaiser-Meyer-Olkin Measure of Sampling Adequacy		0.618
Bartlett׳s Test of Sphericity	Approx. Chi-Square	9010.849
	Degrees of freedom	806
	Significance	0.000

Likert scale as seen in the questionnaire which can be assessed as Supplementary Data 1 in a 5-point scale namely: 1 = strongly disagree, 2 = disagree, 3 = moderately agree, 4 = agree and 5 = strongly agree. Likert scale ranked the perceived factors responsible for the patronage of the traders on the pedestrian bridges using the sum of weighted values (SWV) and average weighted values (AWV). These are shown in Table 8. The factors can be arranged in descending or ascending order in order to fully understand the data, facilitate comparison between the factors or to roughly determine the factors that contributed minimally to the overall average value. The average level of agreed index of the factors responsible for the patronage of the traders on the pedestrian bridge was 2.90 AWV out of an achievable 5. Hence, the factors were moderately agreed.

**Table 8:** Factors responsible for the patronage of the traders on the pedestrian bridges using sum and average weighted values.

**FACTORS**	**OPINION**										**SWV**	**AWV**
	**1**		**2**	**3**		**4**			**5**			
Marketable	4	8			108		304	1250		1674		4.52
Reachable	24	120			252		320	610		1326		3.58
Not stressful	40	104			222		480	420		1266		3.42
Time	8	140			300		600	210		1258		3.4
Satisfactory	40	120			252		400	430		1242		3.36
Distance	8	144			582		304	100		1138		3.08
Availability	50	120			330		440	200		1140		3.08
Safety	48	128			354		384	220		1134		3.06
Attractiveness	48	152			384		280	240		1104		2.98
Convenience	48	168			288		488	100		1092		2.95
Durable	32	180			450		280	140		1082		2.92
Accessibility	62	120			456		328	70		1036		2.8
Handiness	72	120			414		320	100		1026		2.77
Competitive	70	224			240		280	190		1004		2.71
Reliable	88	184			240		248	240		1000		2.7
New items	44	272			252		408	20		996		2.69
Proximity	80	140			396		256	120		992		2.68
Valuable items	76	248			240		200	200		964		2.61
Effectiveness	70	296			222		200	140		928		2.51
Quality of product	132	160			258		176	140		866		2.34
Cost	160	120			210		200	150		840		2.27
Conducive	140	148			270		192	90		840		2.27
Comfortable	152	240			210		112	–		714		1.93

Communality values revealed “not stressful” (60.4%) as the least while”quality of product” (85.3%) had the highest value. Factor analysis finally revealed convenience and effectiveness as factors responsible for pedestrian bridge trading patronage, as perceived by the pedestrians. This can be seen in Table 9 and the factors are arranged in descending order. The result was obtained using the principal component analysis as the extraction method.

**Table 9:** Communalities of variables using principal component analysis as extraction method.

Factors	Initial	Extraction
Quality of product	1	0.853
Proximity	1	0.836
Safety	1	0.835
New items	1	0.832
Effectiveness	1	0.831
Valuable items	1	0.827
Conducive	1	0.819
Satisfactory	1	0.797
Convenience	1	0.791
Durable	1	0.783
Competitive	1	0.78
Time	1	0.772
Reachable	1	0.766
Availability	1	0.762
Accessibility	1	0.757
Marketable	1	0.728
Cost	1	0.718
Attrctiveness	1	0.689
Reliable	1	0.684
Distance	1	0.639
Handiness	1	0.637
Comfortable	1	0.632
Not stressful	1	0.604

The total variance explained is presented in Table 10. As shown in Table 10, all factors that are with Eigen value that are above 1 were extracted and represented under the column extraction sums of square loading. The findings reveal that 10 unconfirmed factors and suggested that there was a cumulative total of 75.54% with the variance of 4.56% and 6.68% at and after extraction which was confirmed after rotational extraction.

**Table 10:** Total Variance Explained of the factors influencing responsible for the patronage of the traders on the pedestrian bridge.

Component	Initial Eigenvalues			Extraction Sums of Squared Loadings			Rotation Sums of Squared Loadings		
	Total	% of Variance	Cumulative %	Total	% of Variance	Cumulative %	Total	% of Variance	Cumulative %
1	2.818	12.251	12.251	2.818	12.251	12.251	2.025	8.804	8.804
2	2.230	9.697	21.948	2.230	9.697	21.948	1.979	8.604	17.408
3	2.099	9.125	31.074	2.099	9.125	31.074	1.846	8.024	25.432
4	1.910	8.303	39.377	1.910	8.303	39.377	1.805	7.848	33.281
5	1.720	7.478	46.855	1.720	7.478	46.855	1.744	7.583	40.864
6	1.590	6.913	53.768	1.590	6.913	53.768	1.661	7.223	48.087
7	1.489	6.473	60.241	1.489	6.473	60.241	1.640	7.131	55.218
8	1.312	5.702	65.943	1.312	5.702	65.943	1.588	6.903	62.120
9	1.159	5.038	70.981	1.159	5.038	70.981	1.549	6.734	68.855
10	1.048	4.558	75.539	1.048	4.558	75.539	1.537	6.684	75.539
11	.951	4.134	79.673						
12	.824	3.582	83.256						
13	.790	3.437	86.692						
14	.623	2.709	89.401						
15	.520	2.259	91.661						
16	.484	2.106	93.766						
17	.425	1.846	95.612						
18	.285	1.237	96.850						
19	.241	1.047	97.896						
20	.154	.671	98.567						
21	.147	.637	99.205						
22	.116	.505	99.710						
23	.067	.290	100.000						

Extraction Method: Principal Component Analysis.

**Fig. 1:**
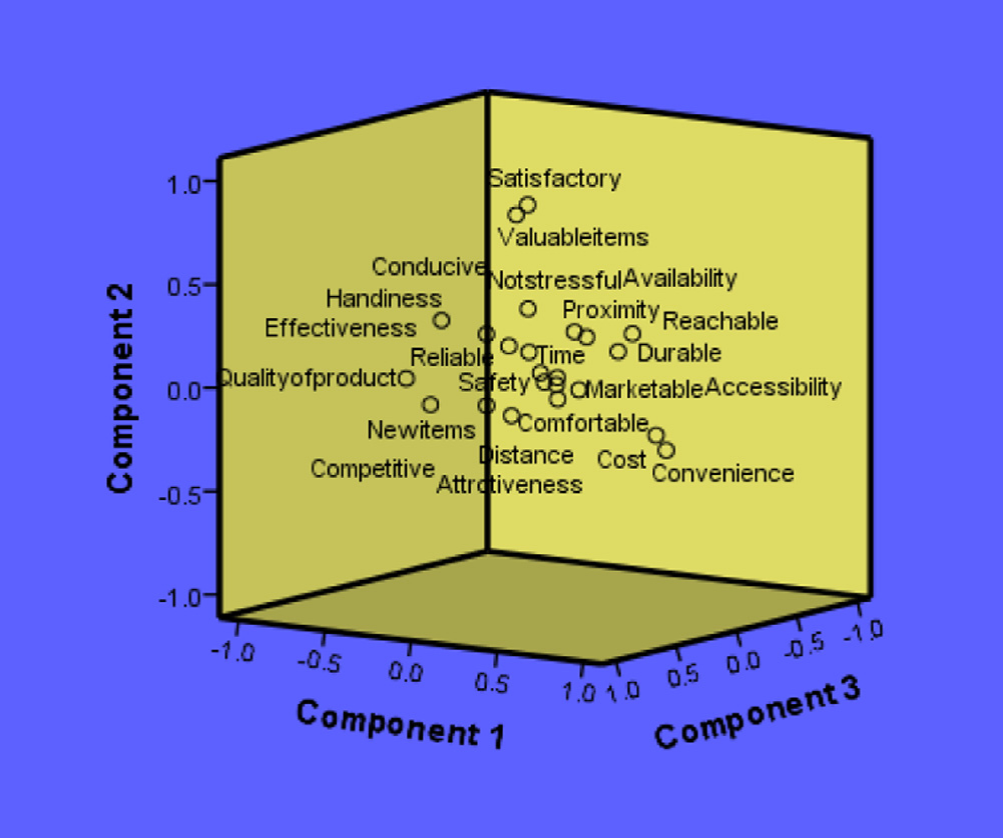
Component plot in rotated space of factors for the patronage of the traders on the pedestrian bridge.

There are various factors responsible for the patronage of the traders on the pedestrian bridge but most reason why the pedestrians patronize the bridge is because of their level of quality, convenience and effectiveness according to the result given by the rotated component matrix as shown in Table 11. Furthermore, component transformation matrix was presented in Table 12 while the summary of the data analysis can be visually seen in Fig. 1. The figure is restricted to first three components with the highest Eigenvalues. However, after various investigations that have been carried out and analyzed, the result of findings shows that there is significant relationship between the socio-economic characteristics of the people using the pedestrian bridge and the factor responsible for the patronage of the traders on the pedestrian bridge. The raw data (set of responses) can be assessed as Supplementary Data 2.

**Table 11:** Rotated Component Matrix of Factors for the patronage of the traders on the pedestrian bridge.

	Component									
	1	2	3	4	5	6	7	8	9	10
Cost	.651	-.211	.009	.431	.147	.009	.158	.102	.009	.085
Distance	.027	.037	.077	-.056	.777	.038	.043	.055	.070	.109
Time	.024	-.007	-.069	-.010	.025	.083	.127	.861	.003	.044
Availability	.057	.205	-.163	-.054	-.133	-.023	.780	-.009	.105	-.222
Quality of product	-.848	-.099	-.061	.156	.191	.120	.013	.110	-.183	.023
Accessibility	-.065	-.068	-.186	.782	-.215	.121	-.121	.074	.019	-.144
Safety	-.042	-.033	-.054	-.886	-.130	-.040	-.086	.035	-.075	-.106
Convenience	.621	-.304	-.119	.020	.210	.227	-.436	.000	.010	-.114
Effectiveness	.162	.331	.715	.130	.233	-.065	.152	.062	-.278	-.065
Handiness	-.460	.261	.200	.105	-.265	.259	-.062	.298	.078	.267
Not stressful	-.038	.342	.086	-.118	.071	-.624	-.089	.005	-.245	.050
Conducive	-.085	.117	.011	.130	.275	.252	.019	-.133	.763	-.206
Reachable	.285	.196	-.318	.107	.023	.078	-.218	.191	.054	-.664
Satisfactory	-.133	.817	-.046	-.104	-.037	.231	-.031	.066	-.020	.198
Durable	.080	.064	-.493	.048	.584	-.016	-.410	-.024	-.123	-.055
Competitive	-.094	-.022	.813	-.175	-.044	.144	-.162	-.061	.163	.005
Valuable items	-.047	.808	.169	.049	.079	-.216	.176	-.117	.095	-.187
Attrctiveness	-.074	-.166	.172	.131	.400	.003	.613	.209	-.028	.170
Reliable	-.071	.175	.197	.098	.122	.754	-.088	.026	-.081	-.010
Proximity	.084	.173	-.225	.059	.157	-.042	-.253	.088	-.020	.804
New items	-.211	-.130	.183	.196	.400	-.365	-.076	.616	.099	-.103
Comfortable	.126	-.080	.074	.080	.158	-.442	.097	-.450	.279	.296
Marketable	.215	-.032	.026	.002	-.130	-.144	.070	.099	.786	.097

Extraction Method: Principal Component Analysis.

Rotation Method: Varimax with Kaiser Normalization.

Rotation converged in 24 iterations.

**Table 12:** Component transformation matrix of Factors for the patronage of the traders on the pedestrian bridge.

Component	1	2	3	4	5	6	7	8	9	10
1	-.606	.510	.442	-.217	-.139	.006	.268	.073	-.114	.143
2	-.221	-.048	.059	.619	.425	.300	.035	.535	.011	.061
3	.364	.058	.400	.156	.311	-.446	.490	-.114	.354	.076
4	-.083	-.125	-.053	-.249	.489	-.438	-.260	.077	-.424	.479
5	.261	.798	-.193	.016	.348	.058	-.286	-.084	-.006	-.209
6	.136	-.109	.566	-.090	.142	.482	-.422	-.305	.183	.289
7	.247	-.152	.377	-.390	.159	.088	.048	.390	-.332	-.569
8	-.254	-.087	-.236	-.505	.291	.047	-.023	.234	.688	-.051
9	.463	.198	-.025	-.145	-.376	.079	.012	.576	.075	.490
10	-.132	.018	.285	.217	-.269	-.518	-.596	.226	.246	-.216

Extraction Method: Principal Component Analysis.

Rotation Method: Varimax with Kaiser Normalization.
