# Supplementary material for: Datasets on factors influencing trading on pedestrian bridges along Ikorodu road, Lagos, Nigeria
Source: Data Brief. 2018 Jun 22;19:1584–93. doi: 10.1016/j.dib.2018.06.055 (PMC6141263; doi:10.1016/j.dib.2018.06.055)
Supplement: Supplementary file 1 — Supplementary material [file mmc1.pdf]

COVENANT UNIVERSITY

COLLEGE OF SCIENCE AND TECHNOLOGY

DEPARTMENT OF MATHEMATICS

CANAANLAND, KM 10, IDIROKO ROAD
P.M.B 1023, OTA, OGUN STATE, NIGERIA
www.covenantuniversity.edu.ng, mat.covenantuniversity.edu.ng

EXTERNAL MEMO

To: Editor, Data in Brief
From: Corresponding Author
Date: 4th June, 2018
Subject: **Conflict of Interest**

I thereby declare the absence of any conflict of interest among the authors.

The authors have read the final draft and unanimously agreed that the paper be sent for review.

Hilary I. Okagbue
Department of Mathematics, College of Science and Technology, Covenant University, Ota.
Google Scholar: *Hilary Izuchukwu Okagbue*
Research Gate: *Hilary Okagbue*
Live DNA: 234.14453
Scopus ID: 56438006100
ORCID: 0000-0002-3779-9763
