# Supplementary material for: Datasets on factors influencing trading on pedestrian bridges along Ikorodu road, Lagos, Nigeria
Source: Data Brief. 2018 Jun 22;19:1584–93. doi: 10.1016/j.dib.2018.06.055 (PMC6141263; doi:10.1016/j.dib.2018.06.055)
Supplement: Supplementary file 2 — Supplementary material [file mmc2.docx]

**(QUESTIONNAIRE)**

**YABA COLLEGE OF TECHNOLOGY, SCHOOL OF ENVIRONMENTAL STUDIES, DEPARTMENT OF URBAN AND REGIONAL PLANNING**

**QUESTIONNAIRE**

**INTRODUCTION:** This questionnaire seeks to elicit information on the assessment of trading on effective use of urban infrastructure in the context of selected pedestrian bridges along ikorodu road, lagos. It is for an academic exercise purpose. Please you are respectfully requested to complete the items as dispassionately as you can. The confidentiality of your responses is assured.

**Section A**

**(Socio-economic features of the people using the pedestrian bridges)**

1. Sex (a) Male (b) Female

2. Age (a) 10-20yrs (b) 21-41yrs (c) 41-60yrs (d ) 61and above

3. Marital status (a) single (b) married (c) divorced (d) widow/widower

4. Religion (a) Christianity (b) Islam (c) African Traditional religion

5. Level of Education Attained (a) Primary (b) Secondary (c) University (d) Informal Training

6. Level of monthly Income (a) Below #10,000 (b) #11,000-#20,000 (c)# 21,000-#30,000

(d) #31,000 and Above

**Section B**

**(What are the pedestrians’ perceived implications of trading on the pedestrian bridge?)**

7. Is there any form of harassment in using the pedestrian bridge? (a) Yes (b) No

8. If yes, is it from (a) Government officials (b) Beggars (c) None (d) Others

9. Have you ever been robbed or molested on the pedestrian bridge? (a) Yes (b) No

10. Awareness of the risk(s) associated with trading on the pedestrian bridge? (a) Yes (b) No

11. Do you consider it save by patronizing the traders on the pedestrian bridge? (a) Yes (b) No

12. How safe is the pedestrian bridge is? (a) Very safe (b) safe (c) partially safe (d) Not safe

13. Opinion about the general hazard caused by people trading on the bridge on their lives.

(a) Very high opinion (b) High opinion (c) Low opinion (d) Very low opinion (e) No response

**Section C**

**(How does pedestrian bridge trading affect movement of people on the bridge?)**

14. How frequent do you use bridge? (a) Often (b) occasionally (c) once in a while (d) everyday

15. How useful is the pedestrian bridges to you? (a) Very useful (b) Useful (c) Partially useful (d) Not useful.

16. How many minutes does it take to walk on the pedestrian bridge? (a) 1-3minutes (b) 4-6 minutes (c) 6-9 minutes (d) over 9 minutes.

17. Would it be faster if the traders are not on the pedestrian bridge? (a) Yes (b) No

18. How does trading on pedestrian bridges affect the ease of movement of people? (a) Partially affected (b) Fully affected (c) Affected (d) Not affected.

19. In your opinion, do they occupy a significant portion of the width of the pedestrian bridge? (a) Yes (b) No

20. What hazard does people trading on the pedestrian bridge cause on urban environment?

(a) Crime (b) Traffic congestion (c) Conflict spatial disorderliness (d) Environmental degradation

21. Do you appreciate the presence of the traders’ on the pedestrian bridge? (a) Yes (b) No

22. What solution will u prefer for the people using the pedestrian bridge for trading?

______________________________________________________________

**SECTION D**

**(What are the factors responsible for the patronage of the traders on the pedestrian bridge?)**

| Level of agreement  variables | Strongly  Disagree | Disagree | Moderately  Agree | Agree | Strongly  Agree |
| --- | --- | --- | --- | --- | --- |
| Cost |  |  |  |  |  |
| Distance |  |  |  |  |  |
| Time |  |  |  |  |  |
| Availability |  |  |  |  |  |
| Quality of product |  |  |  |  |  |
| Accessibility |  |  |  |  |  |
| Safety |  |  |  |  |  |
| Convenience |  |  |  |  |  |
| Effectiveness |  |  |  |  |  |
| Handiness |  |  |  |  |  |
| Not stressful |  |  |  |  |  |
| Conducive |  |  |  |  |  |
| Reachable |  |  |  |  |  |
| Satisfactory |  |  |  |  |  |
| Durable |  |  |  |  |  |
| Competitive |  |  |  |  |  |
| Valuable items |  |  |  |  |  |
| Attractiveness |  |  |  |  |  |
| Reliable |  |  |  |  |  |
| Proximity |  |  |  |  |  |
| New items |  |  |  |  |  |
| Comfortable |  |  |  |  |  |
| Marketable |  |  |  |  |  |
